# Dose-Effect Relationship of Botulinum Toxin Type A in the Management of Strabismus: A Review

**DOI:** 10.7759/cureus.71271

**Published:** 2024-10-11

**Authors:** Asrar L Alhejaili, Aaesha A Alkayyal, Razan A Alawaz, Esraa K Alshareef, Hussain Al-Habboubi

**Affiliations:** 1 Pediatric Ophthalmology, King Abdullah Specialist Children's Hospital, Riyadh, SAU; 2 Internal Medicine, Saudi German Hospital, Medina, SAU; 3 Ophthalmology, Ministry of Health Holdings, Medina, SAU; 4 Ophthalmology, King Salman Medical City, Medina, SAU; 5 Ophthalmology, Prince Mohammed bin Abdulaziz Hospital, National Guard Health Affairs, Medina, SAU

**Keywords:** botulinum toxin type a, esotropia, exotropia, paralytic strabismus, strabismus

## Abstract

Botulinum toxin type A (BTXA) is a well-accepted non-surgical therapy for strabismus. However, its dose and technique vary widely. We reviewed published evidence on the dose effect of BTXA in treating various types of strabismus. Articles on BTXA therapy outcomes were reviewed to study doses and brands of BTXA used and their efficacy for specific types of strabismus. Researchers indicated that greater dosages may be used safely without increasing the risk of complications. The review suggested a positive dose-response relationship of BTX to success but with a higher risk of complications. No single dose of BTX could be recommended for strabismus management.

## Introduction and background

Strabismus, which is misalignment of the eyes, usually starts in early childhood, and its impact on vision-related quality of life, overall development, and personality continues even in adulthood. Early detection and treatment of strabismus in childhood have high success in alignment and binocularity [1-3]. The prevalence of strabismus in children ranged from 2.1% in the UK to 2.6% in Australia [4,5]. The global pooled prevalence of strabismus in all ages was 1.93 (95% CI 1.64-2.21) [6]. Both for the person with strabismus and parents, management of strabismus is crucial for cosmesis and restoration of vision. The nonsurgical orthoptic treatment of strabismus includes optical correction, amblyopia treatment, prismatic correction, and orthoptic exercises [7]. Muscle surgery and intramuscular injection of botulinum toxin (BTA) are the main treatments after initial optic and orthoptic care is provided. Both methods are considered beneficial; however, which is superior is still debatable [8].

Botulinum toxin is a neurotoxin produced by Clostridium botulinum, gram-positive anaerobic bacteria. It has seven serotypes: A, B, C, D, E, F, and G. BT type A (BTXA) is widely used for therapeutic purposes [9]. When injected into the target tissue, BTXA binds to the glycoprotein structures at cholinergic nerve terminals. It causes temporary denervation at the site of injection with minimum systemic effects [10]. BTXA is used in ophthalmologic, gastrointestinal, urologic, orthopedic, dermatologic, dental, secretory, painful, cosmetic, and other conditions [11]. When injected in the overacting muscle of the nondominant eye, the strength of the injected muscle becomes weak for a limited time, the muscle strength of the strabismic eye is rebalanced, and the eye position becomes near parallel.

BTXA is helpful in the motor alignment of strabismic muscles, but multiple injections may be needed. However, it may not help sensory alignment [9]. BTXA is used as a primary treatment to avoid complications related to muscle surgery [10]. BTXA therapy helps reduce the time for anesthesia in muscle surgery for infantile esotropia [11]. In paralytic strabismus, BTXA also helps reduce the angle of deviation, mainly in esodeviation [12]. Thus, there is adequate evidence to support the role of BTA in different types of strabismus, but the issue of the dosage of BTA is still debatable and needs further review.

In this narrative review, we present the dose relationship of BTA in different components of strabismus and outcomes.

## Review

Methods

The research and ethical committee of the Ministry of National Guard, Madinah, Saudi Arabia approved this narrative review article. Since no patient was involved in this research, informed written consent was waived for patients. We abided by the tenets of the Helsinki Declaration. 

**Figure 1:**
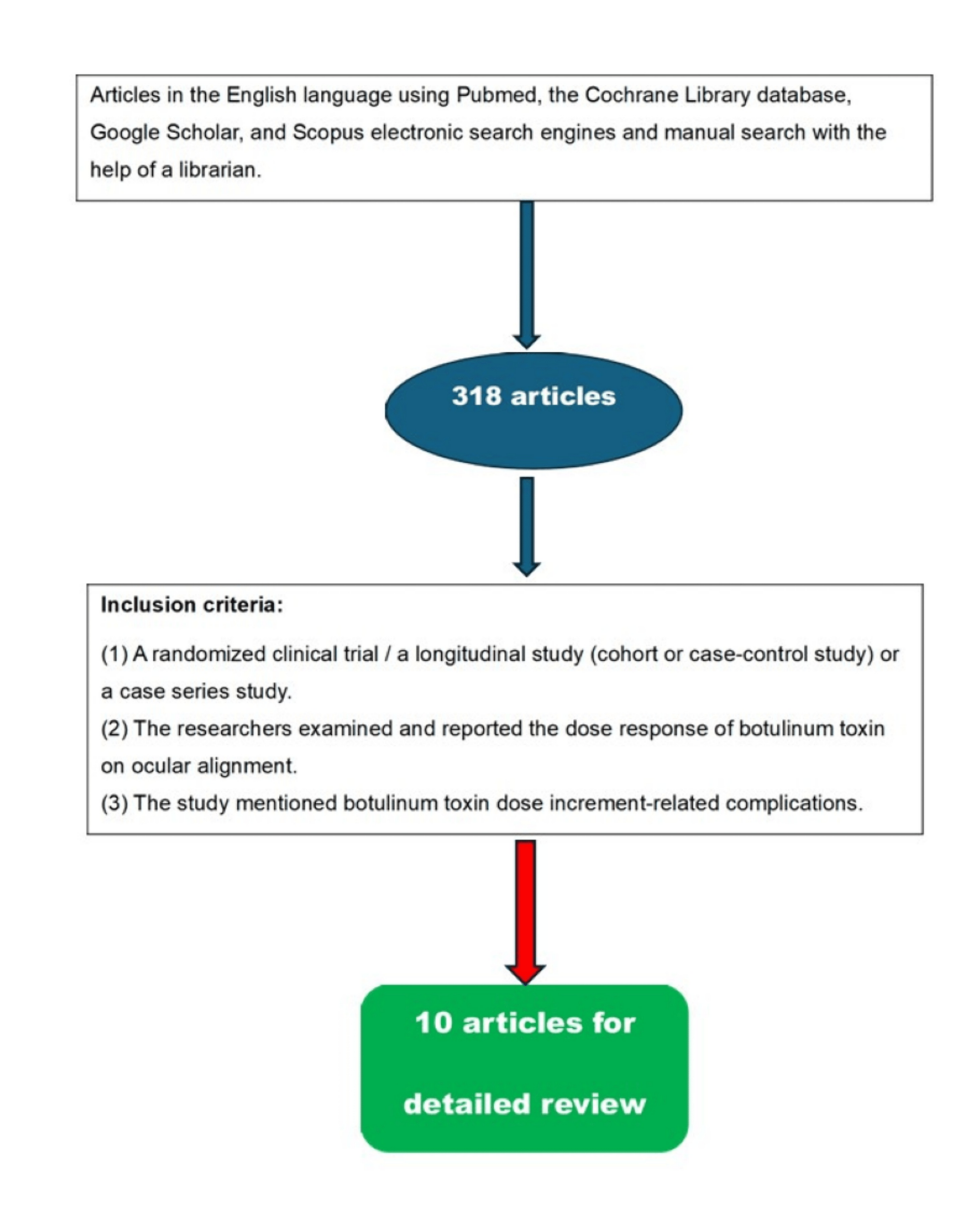
Flow chart displaying the search strategy for research articles on the dose of Botox A for strabismus management

Electronic literature searches were conducted in January 2023 in PubMed, the Cochrane Library database, manual search, and by checking articles reference lists. The search was limited to the studies published in English and without restrictions on publication dates. The search strategy used words like BTXA, botulinum toxin type A, Botox, dose, side effects, complications, strabismus, squint, esotropia, and exotropia (Figure 1).

The search resulted in 318 studies. The abstracts of these studies were reviewed to include the highly relevant studies that met our inclusion criteria, which are as follows: (1) the study was a randomized trial or a comparative study (cohort or case-control study) or a case series study; (2) the study examined and reported the dose-response of botulinum toxin on ocular alignment; (3) the study mentioned botulinum toxin complications that might be related to the dose increments.

Five ophthalmologists were involved in the literature search and manuscript preparation.

To address the observation bias, two investigators' teams independently reviewed the final selected full articles to list the indication, sample size, dose of BTXA, and outcomes, and drug-related side effects and complications were noted. When there was a dispute in reporting by two teams, a senior ophthalmologist provided his input. We also collected information on outcomes in subgroups of known universal confounders like age, gender, and geographic location of procedure.

The procedure for BTXA application is described in detail by Shi et al. and Sener et al. [13,14]. Usually, for a patient who is <14 years old, the procedure is undertaken under general anesthesia. In adult and cooperative children, it is also done using ketamine and topical anesthesia. In the past, BTXA was administered under electromyographic control using a monopolar needle.

In microscopic medial rectus muscle injection of BTXA, the dosage of BTXA has wide variations and is usually based on the angle of deviation before therapy: 1.0 IU~2.5 IU for those < +20△, 2.0 IU ~ 4.0 IU for those from +20△ to +40△, and 4.0 U ~ 6.0 U for those > +40△ [15]. The maximum dosage of each muscle did not exceed 5 IU, and the total injection volume every time did not exceed 0.1 mL. The injection procedure is as follows: (1) The eyelid opener is used to open the eye; 0.5% Alcaine is used for topical anesthesia, and 0.01% epinephrine hydrochloride is used for vasoconstriction; (2) 2% lidocaine hydrochloride is injected for subconjunctival infiltration anesthesia; (3) a conjunctival incision is made near the bulbar conjunctiva on the nasal side of the eye, then the rectus muscle is hooked, and the intermuscular membrane and subconjunctival tissue were separated to expose the muscle fully; (4) the botulinum toxin type A (BTXA) is dissolved in an appropriate amount of sterile saline to configure the required dose of solution; and (5) corresponding dose of BTXA is injected intramuscularly at 5 mm from the muscle insertion (the total amount of each injection should not exceed 0.1 mL).

Results and discussion

Ten articles fit the dose-response relationship of BTXA. The lead author, year of publication, sample details, and intervention, including details of dose, outcomes, and complications are given in Table 1 [14,16,17-24].

**Table 1:** Published articles about the botulinum toxin A injection dose-related outcome of strabismus surgeries EOMS: Extraocular muscle surgery; BTIs: botulinum toxin injections

Author	Year	Study design	Sample	Intervention	Outcome	Complications
Scott [16]	1980	Case series	332 children with primary & operated ET & XT	BTXA -1 injection. (211) 1 to 2.5 units 2 + inj (151) up to 12 units	Ocular alignment (<10 PD) 61%	Transient ptosis 31% - Transient vertical deviation 16%.
Elston et al. [17]	1985	Prospective cohort	85 mean age 40, mean dev 50PD	Low dose BTXA group (3-12 units): (n =44) High dose BTXA group (15.6 units): (n = 21)	Change in deviation after second injection; low dose 52.3% High dose 55%; no deviation; low dose 0%; high dose 9.5%; deviation acceptable with continued treatment; low dose 81.8%; high dose 42.8%	Low dose - need for multiple repeated injections. High dose transient ptosis, and vertical strabismus.
Abbasoglu et al. [18]	1996	Retrospective review	94 patients Pretreatment deviation (mean): ET 17.6+/-0.8 XT -15.9+/-0.8	BTXA dose grs - 2.5U, 5U, 10U, 15U, 20U numbers of injections gr 1, 2, 3, 4.	The average percentage change in initial deviation: ET 46% & XT 29% post-therapy deviation: ET: 10.1 +/- 1.5 degrees XT: -9.9+/-1.3	Ptosis: 14% in XT. 29% in ET.
Sener et al. [14]	2000	Case series	70 patients Mean deviation (PD): ET 38.6 +/- 2.5 XT 37.6 +/- 1.9	BTXA dose: < 35 PD, 2.5 to 7.5 units > 35 PD, 10 to 20 units Injection to single MR or LR 1 to 3 injections, with increasing dose	Successful ocular alignment: ET 32%, XT 22% Acceptable ocular alignment: ET 40%, XT 36%	Transient ptosis 5% in XT. 18% in ET. Transient vertical deviation 27% in XT. 37% in ET.
Ripley et al. [19]	2007	Cohort study	66 patients ET: 39 +XT 27	2 units of Dysport to a single extraocular muscle.	The percentage net change in eso deviation - 52% near and 60% distance. The percentage net change in exo deviation was 60% for near and distance	Ptosis 10.3% in esotropia. 3.7% in exotropia.
Wan et al. [20]	2018	Case series	30 patients Pre-operative deviation: (60-80) Augmented bilateral medial rectus muscle recession	3.7 units per medial rectus muscle with a range of 1.75 to 5 units.	Effect on alignment at BTXA vs only surgery 1 year (5.4 +/- 1.2 vs 3.7 +/- 1.2 PD/mm).	Transient overcorrection 86% Transient ptosis 50%
Handschin et al. [21]	2021	Case series	26 patients (16 BTXA +10 surgery)	BTXA 14.5 IU with a mean dose effect of 1.8 PD/IU.	56% of the BTI and 40% of the EOMS group showed some degree of binocularity	EOMS following BTIs, less surgical dosage
Alshamlan et al. [22]	2021	Prospective cohort	56 patients with esotropia	Gr 1 Eso 11-19 PD BTXA 2.5 IU in 0.05 ml saline per muscle. Gr 2 Eso 20 - 29 PD BTXA 5 IU in 0.1 ml saline Gr 3 Eso 30 - 39 PD and BTXA 7.5 IU in 0.15 ml saline Gr 4 Eso ≥40 PD and BTXA 10 IU in 0.2 ml saline	The angle of deviation ≤10 PD post-therapy Gr 1 75% Overall 39% Lower vs higher dose 57.1 ± 30.4 vs 45 ± 31.4 percentage of improvement in angle deviation	Transient Ptosis, vertical deviation, exotropia
Al Hemaidi et al. [23]	2021	Case series	97 patients of exotropia	BTXA (5–25 IU) per muscle	<10PD in 28.9 % <20PD in 50.5% Basic exo >sensory EX	Transient Ptosis and esotropia
AlShamlan et al. [24]	2022	Case-control study	177 children with infantile esotropia and partially accommodative esotropia (PAET)	BTX group received 100 IU of BTX in 2 ml saline BMR surgery gr	ocular alignment <10 PD of deviation after 1–3 BTX injections success had 1.3 ± 0.6 injections I (1 to 3 injections)	Exotropia in BTXA 0% and 11.8% in surgery gr

In these studies, BTXA was given in dosage from 2.5 IU to as high as 25IU per muscle. The BTXA was given after diluting it with 1 or 2 ml of 0.9% saline. In addition, we also reviewed several articles in which researchers used BTXA in isolation or a combination of other modes of management to manage different components of strabismus. 

Availability of Botox in the Market

Although the present review focused on using BTXA to treat strabismus, we noted different BTXA brands available in the market. Some are used for ocular muscle management, and others for periocular and facial aesthetic treatments (Table 2) [25,26-29].

**Table 2:** Profile of botulinum toxin available in the market to manage strabismus

Generic name	Company	Trade name	FDA approval	Package and dosage	Strengths	Weaknesses
OnabotulinumtoxinA [25]	Allergan, Inc., Irvine, CA, USA	Botox (Global) Vistabel® in Europe and Vistabex® in Italy	Dec 1991	Vials of sterile 50IU, 100IU, 200IU BTXA powder for single use diluted by 0.9% sodium chloride. 0.05-0.15 mL per muscle Administered within 24 hours after reconstitution	Time tested Long-term outcomes known Used in <12 years old, children	The spread of toxins: breathing difficulties, urinary retention, transient ptosis, keratitis due to reduced blinking. Sensitization and reduced efficiency by time.
AbobotulinumtoxinA [26]	Medicis Pharmaceutical Corp., Scottsdale, AZ, USA	Dysport ® Reloxin®/Azzalure®	April 2009			Greater spread effect clinically, leading to a more diffuse distribution of clinical effects
Incobotulinumtoxin [27]	Merz Pharmaceuticals, Frankfurt, Germany	Xeomin	2010	50Iu and 100IU vials.	reduced risk of sensitization or antibody formation	Transient ptosis, bruise, headache
PrabotulinumtoxinA-xvfs [28]	Evolus	Jeuveau	Feb 2019	100IU per vial	Faster onset (within 2-3 days)	High cost, No long-term outcome
OnabotulinumtoxinA [29]	Revance	Daxxify	Sept 2022	100IU per vial	More for facial cosmetic procedures 9 months	Transient ptosis, headache, facial muscle weakness

There is a strong recommendation not to change the brand of BTXA if more than one muscle or repeat of BTXA injection is needed to treat strabismus. 

OnabotulinumtoxinA is supplied in 100-unit and 50-unit single-use vials. It should be reconstituted with sterile, preservative-free 0.9% sodium chloride injection [25].

The dosage for strabismus treatment ranges from 1.25 to 25 units (0.05 and 0.15 mL) per muscle per session. It could be given in one or more than one extraocular muscle. It is noted that a well-developed adult extraocular muscle can accept 3ml of the injected medication. This is a single IM injection because the muscle tissue does not absorb it well in larger volumes. However, the volume injected may need to be adjusted in children and those with less muscle mass [12]. Also, the location of nerve-dense regions differs in different extraocular muscles (EOMs), 51.7% in the medial rectus and 50.1% in the lateral rectus muscle of the length of the muscle belly [30].

Before determining the dose-response relationship of BTXA, it is essential to know that the efficacy of BTXA can be affected if it is not stored correctly, the correct dose is determined, reconstitution of injecting material is not done as per guidelines, and BTXA administration techniques are not adequately followed [31]. Scott described the mode of preparation of BTXA for injection and location of EOMs for injecting this medication as an alternative to strabismus surgery [16]. A BTXA dosing table was proposed to treat infantile esotropia as an adjunct to the surgery for strabismus of different degrees [20].

BTXA for Childhood Esotropia

The increment dose of BTXA to treat childhood esotropia and the success rate of reaching <10ΔD of esotropia without overcorrection that needed additional surgery was described by Alshamlan et al. [22]. The success was noted in children with partially accommodating esotropia (59.1%) and infantile esotropia (29.2%) six months after BTXA therapy. Their achievement of success, angle of deviation, and dose of BTXA are given in Table 3. The need for 2nd BTXA to treat residual angle of deviation of >10ΔD in this study was considered as failure. In strabismus with large angles, repeated BTXA is the accepted norm [32]. Although the risk of surgery under general anesthesia in young children can be avoided by BTXA therapy with minimum systemic complications, even in higher dosages of BTXA, motor alignment could be achieved in a higher proportion. Still, visual rehabilitation is needed in the long term to maintain the achievements and attain sensory alignment of EOMs [12,33].

BTXA in Acute Acquired Comitant Esotropia

Acute acquired comitant esotropia (AACE) is characterized by sudden-onset constant esotropia in all gaze and diplopia. BTXA therapy and surgery have been noted to have similar success rates at 6 and 12 months after BTXA treatment or surgery [34]. The injection dose of BTXA was 3.5 units for patients with a deviation angle less than 25 ΔD and 4.0 units for an angle greater than 25 ΔD. In all AACE cases, diplopia disappeared post-injection, and the angle of deviation for both near and distance was significantly reduced [35]. The dose of BTXA to treat AACE seems to vary depending on the angle of deviation before therapy; the one-year success is promising, but additional BTXA may be needed after two years.

BTXA in More Than One Muscle for Esotropia

For treating horizontal strabismus of different types, BTXA is injected in more than one muscle so that the effect is adequate and the amount of medication is divided. The success in esotropia is more conclusive and long-lasting compared to the treatment of exotropia, and it points to muscle spasms as the root cause of strabismus. In 27 patients with near-work-related acquired esotropia, BTXA (BTXA; Hengli, Lanzhou Institute of Biological Products) was injected in the medial rectus of both eyes. After 10 months, all eyes were aligned, and diplopia while reading for a long time was resolved [36]. In the treatment of infantile esotropia, Alam et al. injected BTXA in both medial recti to treat infantile esotropia in 63 infants. The BTXA dose for the first injection in 42 patients was 7 ± 3 IUS, 2nd injection was 6 ± 3 IU in 17 patients, and the 3rd injection was 6 ± 2 IU in four patients. The success rate was 33%, 38%, and 75% after 1st, 2nd, and 3rd injections [37].

BTXA as an Adjunct to Strabismus Surgery

BTXA is injected concurrently in the recessed muscle of non-fixating eyes if the angle of the strabismus is more than 40ΔD before therapy. Tugcu et al., in a small series of 13 patients with exotropia (eight cases) and esotropia (five cases), injected 5IU BTXA and monocular resection recession surgery for large-angle sensory strabismus. The success of achieving <10ΔD after four years was 87.5% in exotropia and 80% in esotropia [38]. Wan et al. noted a positive and significant correlation between BTXA dosage and success in alignment after four months in children with large-angled infantile esotropia who had undergone strabismus surgery and concurrent use of BTXA [20]. In a review by Dashtevska et al., the success of BTXA along with surgery was better than only BTXA as the primary treatment of strabismus. The dose recommended was 2.5 to 5 IU for adjuvant therapy. BTXA was found to be a better alternative to treat residual strabismus following the first surgery instead of subjecting the child to repeat surgery [39].

BTXA for Intermittent Esotropia

The success of BTXA therapy in intermittent esotropia is debatable compared to surgery for large angle deviations [40]. The success of motor alignment (65.7%) was higher in eyes with a lower angle of deviation before BTXA therapy [41]. The dose of BTXA is higher in patients with large-angle intermittent exotropia than those with low-angle intermittent exotropia. Thus, it is possible that a dose of BTXA could also have an indirect influence on successful outcomes.

BTXA for Infantile Esotropia

Most children with infantile esotropia have a large angle of inward deviation [42]. Treatment before six months is recommended to enhance the sensory and cortical devilment of the child [43]. To avoid general anesthesia in young infants, BTXA is an alternative to treat infantile esotropia. However, Mehner et al., in their Cochrane review, reported inconclusive evidence about the benefits of BTXA over the surgery [44]. Therefore, BTXA is recommended as an adjunct therapy to strabismus surgery [45].

BTXA for Congenital Esotropia

To treat congenital esotropia, BTXA was injected into the belly of the muscle using an operating microscope. The dose was calculated using 1 International Unit for ten prism diopters deviation. In an extensive series of 837 children with congenital esotropia with esotropia ranging from 10 to 50 ΔD prism dioptre, 33% had 10 to 20 ΔD, 50% had 21-30 ΔD, and 17% had s>30 ΔD of eso deviation. The dose of BTXA was given in equal amounts in both medial recti in one sitting in 12% of children. The same steps were followed when the injection was repeated. The second injection was given in 18% of cases. The remaining 60% of the children were operated on for extraocular muscle surgeries in addition to two BTXA injections [46].

BTXA for Esotropia Secondary to Paralysis of the Lateral Rectus

The abducent nerve palsy causes AACE. The conventional surgery to treat a stable AACE is medial rectus recession and lateral rectus resection in the affected eye. 5 IU per 0.1 mL of BTXA was injected into the belly of the medial rectus of the deviating eye. Six months after the first injection, the improvement of the angle of deviation was similar at 1 and 3 months but was significantly better in eye operation than when treated with BTXA [47]. A study in Korea showed BTXA as a promising alternative to surgery in managing diplopia following the 6th nerve palsy [48]. BTXA therapy could be an alternative to surgery if the patients' systemic condition does not permit surgery.

BTXA for Esotropia With Oblique Muscle Affection

5IU BTXA is applied to the inferior oblique muscle to address V-pattern strabismus and primary position hypertropia. This was with surgery for horizontal muscles. Oke et al., in their study of 31 IQ muscle therapy of 20 patients, concluded that it has short-term benefits in treating hypertropia in the primary position. Multiple injections and less efficient long-term results favored surgical management [49]. In another study, four IU of BTXA were administered in the IO of 21 patients with superior oblique muscle palsy. Hypertropia was resolved, and the patient perceived an improved quality of life six months after treatment, which was also promising [50].

BTXA for Exotropia

Among children undergoing chemodenervation for strabismus at a tertiary eye hospital in the UK, 10% were with exotropia. Thus, BTXA therapy is more common to treat esotropia than exotropia. Dysport® 2.5 units (in 0.1ml of normal saline) were used for each lateral rectus muscle. The reduction in the deviation in size was 80% four months after the BTXA injection [30]. In a study of 97 exotropia managed by BTXA in a tertiary eye hospital in Riyadh, Saudi Arabia, authors reported 54 cases who were given <20 IU of BTXA; the success (<10ΔD of exodeviation) could be achieved in 16.7%. While in 43 cases that were given >20 units of BTXA, the success rate was 44.2% [23]. There seems to be a relationship between the dose of BTXA and the outcome of therapy for exotropia. However, the exotropia subgroups in these two studies were too small to conclude the dose-response relationship in subgroups.

BTXA for Intermittent Exotropia

In a study of 72 cases of intermittent exotropia, two different treatment doses were given depending on the severity of strabismus. BTXA was injected 1.25 IU per muscle in patients with strabismus ≥ − 15 ΔD and < − 20 ΔD, while 2.5 IU per muscle was injected in patients with strabismus ≥ − 20 ΔD and < − 50 ΔD. But had exotropia of more than 20 and were given 2.5IU, and the success rate of achieving <10 ΔD was 52.8% after six months [51]. In a review, Joyce et al. noted that BTXA, irrespective of the dose, seems less effective in improving the angle of deviation; they are rarely associated with adverse outcomes [52]. Razavi et al. used 10IU of AbobotulinumtoxinA injected in both lateral rectus muscles of patients with intermittent exotropia. The treatment age and six months of follow-up influenced the success of intermittent exotropia treatment by BTXA, although effective for achieving angles 10 to 20 ΔD [53].

BTXA in Syndromic Strabismus

In addition to Down syndrome and cerebral palsy, several syndromes are known to be associated with strabismus. They include Apert syndrome, Congenital rubella, Hemangioma near the eye during infancy, Incontinentia pigmenti syndrome, Noonan syndrome, and Prader-Willi syndrome. In adult graves disease, Guillain Barrie syndrome, and diabetes can also cause strabismus [54]. Many cause horizontal and vertical muscle movement anomalies, resulting in A and V syndromes [55].

When treated with BTXA injected into horizontal muscles, people with Duane Retraction Syndrome could improve their head posture and quality of life [56]. Treating and locating the muscle responsible for manifestation is often challenging [57]. Thus, how many muscles are to be injected and what dosage of BTXA is still a matter of debate. There is no natural dose-response curve for BTXA injection. Ozkan et al. suggested 5IU as the standard dose but recommended reducing the dose to 2.5IU in infants and increasing the dose up to 10 U in thyroid orbitopathy [58].

Refractive Correction and Orthoptics to Evaluate the BTXA Dose Response

The goal of achieving <10ΔD deviation and stereopsis after BTXA must be evaluated after correction of refractive error and compliance post injection so that parameters related to deviation at near and distance, accommodation, and convergence can be assessed for short as well as long term, especially in horizontal deviations mainly esotropia [59]. Refractive correction and compliance with the recommended management mode, including surgery and BTXA, are crucial for success and, therefore, are part of the preferred pattern of Practice for strabismus management [8].

Complications and Higher Doses of BTXA

All botulinum toxin products may spread from the injection area to produce symptoms consistent with botulinum toxin effects. These may include asthenia, generalized muscle weakness, diplopia, ptosis, dysphagia, dysphonia, dysarthria, urinary incontinence, and breathing difficulties. These symptoms have been reported hours to weeks after injection. Swallowing and breathing difficulties can be life-threatening, and there have been reports of death. The risk of symptoms is probably most significant in children treated for spasticity. Still, symptoms can also occur in adults treated for spasticity and other conditions, particularly in patients with an underlying condition predisposing them to these symptoms. In unapproved uses, including spasticity in children, and approved indications, cases of spread of effect have been reported at doses comparable to those used to treat cervical dystonia and spasticity and at lower doses [60]. Transient ptosis, vertical deviation, and subconjunctival hemorrhage are local transient side effects noted but mainly based on the volume of injected material and precautions taken to avoid dissipation and accidentally injuring the blood vessels [61]. The use of higher doses of BTXA resulted in more severe but transient ptosis [22]. In three cases, tonic pupils were reported after injecting 3 to 6 IU BTXA per muscle in strabismus management [62].

In epidemiology, establishing a dose-response relationship between risk factors and outcomes is essential for establishing a causal association [63]. However, it is not always linear. Multiple effect modifiers influence the outcome simultaneously, resulting in a non-linear response curve. Mathematicians and statisticians find it challenging to explain such a relationship [64]. More work is recommended on the dose-response relationship of BTXA to the reduction of motor deviation and sensory alignment in strabismus. 

After optical and orthoptic care for persons with strabismus, surgery, and neurodenervation by BTXA are two options for strabismologist. The dose of BTXA is not standard and varies based on the type of strabismus, angle of deviation, primary vs concurrent surgery, and age of the patient. Therefore, the surgeon's experience could also influence the decision to start at a low dose and adjust the dose if required instead of providing a higher dose of BTXA, as there is a risk of over-correction and transient side effects like ptosis. Counseling of parents of children with strabismus with a higher angle of deviation to be treated with BTXA should offer caution about the possibility of under-correction and the need for further injections.

## Conclusions

Several brands of BTXA are available on the market. Not all are useful for treating strabismus. As the dosage increased, efficiency increased, but complications also increased. The effect of BTXA is for a limited period, and repeated injections may be needed to correct large angles of strabismus. The review has shown that the dose of BTXA to treat strabismus is still a matter of research and is linked to establishing the benefit of BTXA over surgery. The caregiver should consider the type of BTXA, strabismus type, the angle of deviation, primary treatment or an adjunct to concurrent surgery, and the patient's age.
